# An action research study of quality improvement in instrument packaging procedures for the central sterile supply department

**DOI:** 10.1038/s41598-024-54237-z

**Published:** 2024-02-14

**Authors:** Wei Pan, Liangying Yi, Ting Hu, Juanli Huang, Yongdeng Huang

**Affiliations:** 1 Department of Sterile Processing Nursing, West China Second University Hospital, Sichuan University, Chengdu, Sichuan China; 2 Key Laboratory of Birth Defects and Related Diseases of Women and Children (Sichuan University), Ministry of Education, Chengdu, Sichuan China

**Keywords:** Surgical instruments, Product packaging, Equipment failure, Patient safety, Health care, Risk factors

## Abstract

This study aimed to reduce instrument packaging defects in the Central Sterile Supply Department (CSSD) using action research. Data of the instrument packs packaged by the packaging personnel at the CSSD of the authors’ institution during March to May 2023 were collected and analyzed. After identifying the problems, 2 rounds of cyclic process of “plan-action-observe-reflect” were implemented to standardize the packaging procedures and develop and improve the applicable check of standard operating procedures for the CSSD. After strictly implementing the packaging operation standards and checklists, the number of packaging defect cases dropped from 274 to 41. A significant difference was identified between the number of packaging personnel who achieved a “pass” in the assessment of 3 items for maintenance. Also, 1 item for assembly had significant differences compared with the baseline number after the first cycle (*P* ≤ 0.001). A significant difference was identified between the number of packaging personnel who achieved a “pass” in the assessment of 20 items for 6 components after the second cycle compared with that after the first cycle (*P* ≤ 0.05). Through action research methodology, strict implementation of standardized packaging procedures in the CSSD can reduce packaging defects, thereby decreasing clinical complaints and ensuring patient safety.

## Introduction

Packaging is important in handling all reusable diagnostic and therapeutic instruments, tools, and items. It mainly includes assembly, packaging, sealing, and labeling steps. Packaging defects can occur due to various reasons, e.g., failure to strictly follow packaging procedures, lack of knowledge about instrument packaging, model mismatch due to careless mistakes in check, instruments that are not thoroughly cleaned, and quantity errors. Complete sets of instruments that need to be assembled are commonly packaged using non-woven fabrics, or rigid containers for closed-type packaging, which makes users unable to check instruments before unpacking. Quality control of packaging is the premise of ensuring the high quality of the instrument packs. Implementing clinical diagnostic and therapeutic activities is inextricably related to the quality of the instrument packs. Instrument packs with defects can cause disruptions in diagnostic and therapeutic activities, affecting the quality and efficiency of clinical healthcare professionals’ work. These defects pose risks to patient safety^1^ and can lead to medical disputes. Packaging defects for the Central Sterile Supply Department (CSSD) increase personnel, finances, and resource costs and reduce satisfaction with high-quality services. There is relevant research on packaging defects worldwide. These include using the quality control circle, root cause analysis and the medical failure mode to reduce the incidence of packaging defects^2–4^. However, these studies lack close communication and co-operation between researchers and practitioners. They also involve simple implementation of research objects subject to the countermeasures stimulated by researchers and managers after discussion. Action research is a method that closely integrates research with solving practical problems at work. It involves a small-scale intervention in real-world activities and carefully assesses the impact of such intervention^5^. Action research aims to solve problems through co-operative self-reflection based on critical theory. It focuses more on changing a current situation, attaches importance to practice, and emphasizes that researchers and practitioners are in the same position^5^. For instrument packaging, which is practical work, managers hope packaging personnel work in strict accordance with guidelines and procedures. However, managers rarely work with packaging personnel to jointly find out and communicate problems in order to achieve the purpose of changing the current situation. Action research can remedy this kind of situation. It not only requires practitioners to implement their work strictly, as required to avoid mistakes, but it can also help to find the reasons for defect occurrence through reflection and communication. Action research has been widely applied in nursing research abroad^6^, and in recent years, it has been extensively used in nursing education and clinical practice in China^7–10^, but the studies of action research in work procedures for the CSSD are rare. This study aimed to reduce packaging defects during instrument packaging in the CSSD using action research. Following the 3 months of action research (problems being identified and instrument packaging defects being clarified in March 2023; and 2 rounds of research circle (plan-action-observe-reflect) being implemented from April to May 2023), the number of packaging defects was significantly reduced.

## Methods

### Ethics approval and consent to participate

This study was conducted in accordance with the Declaration of Helsinki. All research methods were carried out in accordance with the relevant guidelines and regulations. This study was approved by the Medical Ethics Committee of West China Second University Hospital, Sichuan University [2023 Medical Scientific Research for Ethical Approval No. (031)].Verbal informed consent was received from all participants. The Medical Ethics Committee of West China Second University Hospital, Sichuan University approved the procedure of obtaining verbal informed consent.

### Packaging personnel

Inclusion Criteria: Nurses and workers who had worked at the packaging posts in our CSSD for at least 6 months. Exclusion Criteria: Leave duration ≥ 3 months; interns and trainees. A total of 52 packaging personnel were included in this study.

### Instrument packs

Inclusion criteria for instrument packs: A complete set of surgical instruments in closed packaging, with a minimum quantity of 15 items. Exclusion criteria for instrument packs: instruments processed by medical device companies or CSSD of other hospital, or a complete set of instruments processed for other hospitals.

Our regular observation on monthly packaging quality inspection results showed that the packing defect rate was 3.6%, and the defect rate dropped to 1.2% after the implementation of supervision and rectification measures. The PASS software was used to calculate the sample size. A sample size of 828 cases needed at different research stages was determined by 80% power and 5% level of significance. Considering the deletion and loss of samples, a sample size of 900 instrument packs were needed at different research stages in this study.

### Establishment of the research team

The research team consisted of the author WP, 1 department-level head nurse, 2 other head nurse, and 2 nurses. WP and the head nurses were nursing experts at the Chinese nursing hierarchy of CN4, and the 2 nurses were supervising nurses at the Chinese nursing hierarchy of CN3. The research team members were instructors and examiners in the research. The action team consisted of 52 packaging personnel, including 32 workers and 20 nurses, who were mainly responsible for packaging inspection and feedback on problems. WP gave unified training to the research team members on the details of packaging and assessment criteria. The training duration was 1 week. Training content included packaging procedures, inspection methods of instruments, standards of the quality and function of instrument cleaning, the methods of instrument assembly, package sealing standards and the content of the check. WP conducted demonstration and assessments for the research team members. The assessment criteria was subject to the “Central Sterile Supply Department Closed-Type Packaging Operation Assessment Criteria” and the “Instrument Packaging Checklists”.

### Action research method

The action research method is adopted to identify the issues and clarify the defects during the instrument and item packaging process. Packaging flaws are minimized using the iterative process of “plan-action-observe-reflect”. The specific methods are as follows:

#### Problem identification and situation analysis

Two quality control nurses from the team examined the instrument assembly and packaging performed by packaging personnel during March 2023. They identified a total of 274 packaging defects, including 73 cases of instrument quantity defects, 20 cases of unclear or missing important information on external labeling, 42 cases of chemical indicator cards not placed in high-risk instrument packages, 10 cases of misplaced indicator cards, 65 cases of unrecognized inadequate cleaning quality of instruments, 18 cases of unrecognized instrument functional damage, 22 cases of model mismatches, and 24 cases of substandard sealing (loose packaging).

In this study, quantity error refers to incorrect quantity of instruments in the pack; model matching defect refers to assembling instruments mismatched, such as the mismatch between hand shank and sheath; cleaning quality defect refers to dirt, blood stains and tissue residue being found on the surface, teeth and joint of the instrument; functional defect refers to imprecise or misaligned occlusion in the functional parts of the instrument; chemical indicator defect refers to missing chemical indicator in the pack or an incorrect type of chemical indicator being placed in the pack; sealing defect refers to loose seal; and labeling defect refers to indiscernible or incomplete information in the label.

Based on the defect analysis and actual work situation, the research team identified possible causes for these issues, including failure to strictly follow packaging procedures, lack of knowledge about instrument packaging, lack of attention to detail due to heavy workload, presence of environmental factors causing interference, and unclear item classification leading to disorganized placement, and other factors.

#### Developing a plan

**Figure 1:**
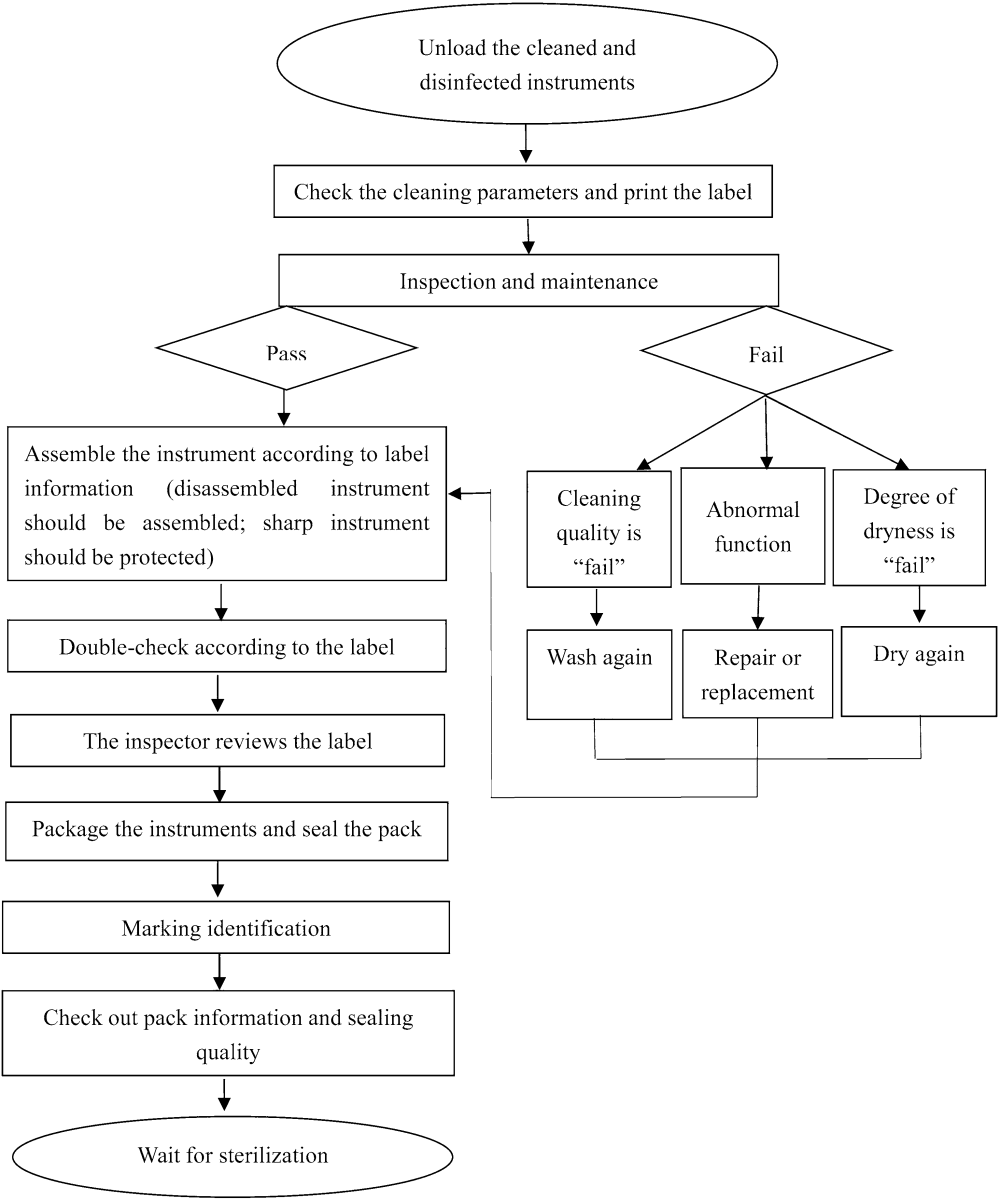
Instrument packaging procedures.

Methods such as situational analysis, group discussions, and literature review were employed to determine improvement strategies and measures. The plan consists of four aspects:

- *Institutional procedures*. Revise and refine the packaging system processes, establish a comprehensive packaging operation procedure, and create a standard operating procedure (SOP) to provide packaging personnel with standardized references. Figure 1 depicts the packaging process.
- *Standardized instrument mapping*. Develop standardized instrument diagrams depending on clinical requirements and instrument types. These diagrams should include instrument details, configuration processes, placement order, and specifications and quantity requirements. This will allow packaging personnel, especially those recently assigned, to properly comprehend instruments and packaging requirements and perform operations based on the provided diagrams.
- *Training and assessment*. Develop a training strategy for packaging skills and standard instructional videos for skill training to ensure standardized training procedures. Regular assessments should be conducted for personnel at all levels, and a packaging skills competition should be organized to motivate employees and solidify their understanding of packaging skills.
- *Establishing a robust dual-verification system*. Implement a dual-check system after assembly but before packaging to confirm the accuracy of instrument types, models, and quantities.

#### Implementation plan

The research team conducted intensive training for the action team members according to the revised packaging rules and procedures. The training lasted for 1 week. The training content included the interpretation of the rules and procedures, cleaning quality standards, functional qualification standards and assembly standards. The training was conducted through demonstration and watching videos about standard packaging operations. At the end of the training, an online theoretical examination was conducted. A score of ≥ 90 meant “pass”. In addition, an operation assessment was conducted using the “Standard Procedures for Closed-Type Packaging”. A score of ≥ 90 meant “pass”.

After the action team members passed the assessments, they strictly implemented the packaging procedures and carried out packaging with the help of the instrument diagrams. WP and the 3 head nurses went to the work site to provide guidance to the packaging personnel on packaging and execution during April 2023 and May 2023. They gave guidance and supervision for a total of 2 cycles within 2 months, namely 1 cycle per month. The research team members used the “Standard Procedures for Instrument Packaging” and the “Instrument Packaging Checklists” to evaluate the packaging personnel’s implementation of packaging procedures in the last week at the end of each cycle. For assessing the packaging personnel’s knowledge of instrument maintenance, the packaging personnel were evaluated in the last week at the end of each cycle using the “Central Sterile Supply Department Closed-Type Packaging Operation Assessment Criteria”. The researchers collected the problems encountered by packaging personnel at work, along with their suggestions, in order to improve the packaging procedures.

#### Observation and reflection

Observation involves examining and documenting the process as well as the outcome of actions. It is an essential prerequisite for data collection in action research^11^. During the execution and verification process of the packaging procedure, researchers actively engage with packaging personnel, gaining insights into the execution of the process and inquiring about any encountered issues apart from providing on-site guidance. There are 4 packaging posts in the CSSD. The researchers observed the rotating staff in the 4 packaging posts at work and recorded the defects in the defect registration form. This form contained the following information: name of the instrument package, defect description (including defects on quantity of instruments, cleaning quality, instrument function, model matching, chemical indicator, sealing, and label information), check execution, adherence to the standard packaging procedures, date and time of packaging, and name of the packaging person. Defects on cleaning quality, instrument function and model should be detailed to the exact part of each instrument (such as groove,joint, and hand lever). The researchers conducted a statistical analysis and comparison on the implementation of packaging procedures after each round of actions, and discussed problems with the action team members.

Following the first cycle of actions, packaging personnel become proficient in the packaging procedure and carry it out accordingly, resulting in a decrease in the number of packaging defects compared to before the actions were conducted. Based on the on-site inspections, guidance processes, discussions with the action team members, and reflections, the research team collectively summarizes the observed and encountered challenges in packaging practice as follows:

- During busy periods, limited personnel are available for dual verification and quality of check is poor, leading to extended working hours.
- Improper cleaning and classification result in the mixing of instruments from different departments. Each packaging station needs to perform secondary classification, as errors in instrument types are prone to occur.
- Excessive items on the packaging workbench impede the packaging process and contribute to quantity errors.

The proposed solutions are as follows:

- Implement 6S management to maintain a clean and clutter-free packaging workbench. Packaging materials should be organized in drawers, following the easy access principle.
- Perform instrument cleaning based on departmental collection and categorization. The name of the department should be written on each frame, and instruments from different departments should not be mixed.
- Establish a flexible scheduling system to adjust staffing during increased workload, ensuring that packaging verification is done as specified.

After implementing the above solutions to address the highlighted issues in the first cycle, the second cycle was implemented in January 2023.

### Evaluation method

Compared to the execution status of instrument packaging processes before, 1 month after, and 2 months after the implementation of action research, the number of defects were observed in terms of quantity, model, cleaning quality, functionality, chemical indicator cards, packaging methods, and label information of items inside the sterilization pouches. Packaging procedures were subject to the “Standard Procedures for Instrument Packaging” and the “Instrument Packaging Checklists”. Packaging defects were identified using the standards for instrument cleaning quality, function, chemical indicators, sealing and labeling.

### Statistical methods

Statistical software SPSS 21.0 was used for statistics; the survey data were statistically described, expressed the standard deviation of the mean using the measurement data, and analyzed the effect before and after the training using χ^2^ test, where *P* < 0.05 was considered significant. Data entry was double-checked. Data were randomly checked to ensure data accuracy and completeness.

## Results

### Demographic information of packaging personnel

Our study included 52 packaging personnel. Details of the packaging personnel are presented in Table 1.

**Table 1:** General information of packaging personnel (n = 52).

Item	n	Percentage (%)
Gender		
Male	16	30.8
Female	36	69.2
Categories of personnel		
Worker	32	61.5
Nurse	20	38.5
Age (years)		
18–25	2	3.8
26–30	13	25
31–40	22	42.3
41–50	8	15.4
> 51	7	13.5
Education background		
Junior high school or below	13	25
Senior high school/vocational school/technical school	17	32.7
College diploma	3	5.8
Bachelor’s degree	17	32.7
Master’s degree and above	2	3.8
Work experience		
1–3 years	12	23.1
4–6 years	20	38.5
7–10 years	12	23.1
> 10 years	8	15.3
Nursing title		
Chief nursing officer	7	35
Nurse practitioner	12	60
Nurse	1	5

### Pareto analysis of packaging defects before action

**Figure 2:**
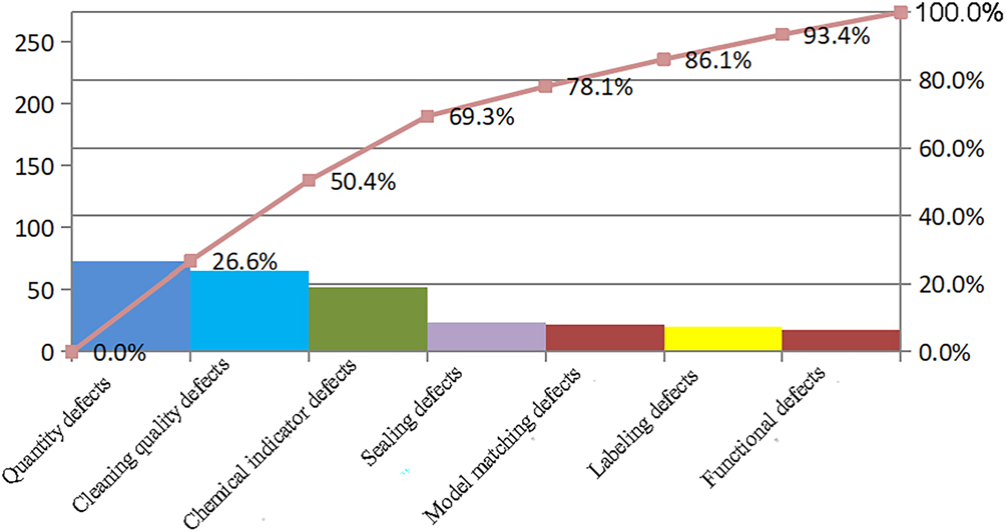
Pareto analysis of packaging defects before action.

Before action, 73 instrument quantity defect cases, 65 cleaning quality defect cases, 52 chemical indicator defect cases, 24 sealing defect cases, 22 instrument model matching defect cases, 20 labeling defect cases, and 18 functional defect cases were identified. The cases of quantity defect, cleaning quality defect, chemical indicator defect and sealing defect accounted for 80% of the instrument packs with packaging defects. Details are presented in Fig. 2.

### Incidences of instrument packaging defects before and after action

The incidence of packaging defects following action was significantly reduced compared with that before action. After action, the number of instrument quantity defect cases decreased from 73 to 15; the number of model matching defect cases decreased from 22 to 2; the number of cleaning quality defect cases decreased from 65 to 15; the number of instrument functional defect cases decreased from 18 to 0; the number of chemical indicator defect cases decreased from 52 to 5; the number of sealing defect cases decreased from 24 to 4; and the number of labeling defect cases decreased from 20 to 0. Statistically significant differences were identified by *P* < 0.05. Details are shown in Table 2.

**Table 2:** Number of cases of instrument packaging defects in different research stages.

Stage	Number of cases	Quantity defects	Model matching defects	Cleaning quality defects	Functional defects	Chemical indicator defects		Sealing defects	Labeling defects
						Not put in	Misplacement		
Before action	900	73	22	65	18	42	10	24	20
Action cycle 1	900	45	10	40	10	18	3	14	10
Action cycle 2	900	15	2	15	0	5	0	4	0
χ^2^		39.921	18.110	32.703	17.611	33.325	12.213	14.511	20.225
*P*		< 0.001	< 0.001	< 0.001	< 0.001	< 0.001	0.002	0.001	< 0.001

### Implementation of packaging by action team members

The research team refined and amended the content of the packaging procedures to clarify the content that packaging personnel should perform in their work, including the details of maintenance and check. A significant difference was identified between the number of packaging personnel who achieved a “pass”in the assessment of 3 items for maintenance. Also, 1 item for assembly had significant differences compared with the baseline number after the first cycle (*P* ≤ 0.001). A significant difference was identified between the number of packaging personnel who achieved a “pass” in the assessment of 20 items for 6 components after the second cycle and for that after the first cycle (*P* ≤ 0.05). Details are presented in Table 3.

**Table 3:** Comparison of the pass rates of the packaging process performed by the action personnel in different research stages.

Item	Baseline		Action cycle 1		X^2^	*P*	Action cycle 2		X^2^	*P*
	n	Pass (%)	n	Pass (%)			n	Pass (%)		
Preparation before packaging										
1. Wear heat-resistant gloves and unload instruments that have been cleaned and disinfected after hand sanitization	52	28 (53.85)	52	36 (69.23)	2.600	0.107	52	50 (96.15)	24.821	< 0.001
2. Review the cleaning process, cross-check the instrument types and quantities using the recovery records, and print out traceability labels	52	15 (28.85)	52	28 (53.85)	6.701	0.01	52	52 (100.0)	57.433	< 0.001
3. Prepare perforated instrument trays and line them with absorbent paper	52	52 (100.0)	52	52 (100.0)	–		52	52 (100.0)	–	
4. Place a chemical indicator card inside the package	52	28 (53.85)	52	40 (76.92)	6.188	0.013	52	49 (94.23)	22.061	< 0.001
Inspection and maintenance										
1. Check for missing parts by comparing them with the instrument labels	52	24 (46.15)	52	38 (73.07)	7.828	0.005	52	50 (96.15)	31.668	< 0.001
2. Examine the cleanliness and dryness of the instrument	52	38 (73.08)	52	44 (84.62)	2.075	0.15	52	52 (100.0)	16.178	< 0.001
3. Check for structural damage and ensure that all instrument components are complete and intact	52	15 (28.85)	52	32 (61.54)	11.219	0.001	52	48 (92.31)	43.847	< 0.001
4. Verify that the instrument joints are flexible but not loose, that the instrument tips close precisely without gaps, and movable joints are lubricated	52	12 (23.08)	52	35 (67.31)	20.536	< 0.001	52	47 (90.38)	47.985	< 0.001
5. Examine the instruments for missing rivets, screws, and other fasteners at the joints and fixation points	52	10 (19.23)	52	32 (61.54)	19.330	< 0.001	52	49 (94.23)	59.580	< 0.001
6. Check that sharp instruments have sharp functional ends without rolled edges	52	21 (40.38)	52	44 (84.62)	21.703	< 0.001	52	52 (100.0)	44.164	< 0.001
7. Ensure that all lumens are clear, and check rubber tubing for signs of aging, adhesion, or cracks	52	40 (76.92)	52	49 (94.23)	6.310	0.012	52	52 (100.0)	13.565	< 0.001
Assembly										
1. Use the appropriate assembly techniques specified in the instrument assembly procedure for different types of instruments	52	20 (38.46)	52	40 (76.92)	15.758	< 0.001	52	51 (98.08)	42.656	< 0.001
2. Ensure that the diameter of wrapped pipes and power cables is greater than 10 cm	52	33 (63.46)	52	41 (78.85)	2.998	0.083	52	52 (100.0)	23.247	< 0.002
3. Keep all cavities and valves open, and use safety coverings on any sensitive equipment and sharp objects	52	39 (75.0)	52	45 (86.53)	2.229	0.135	52	52 (100.0)	14.857	< 0.003
4. Arrange the instruments according to the principles of instrument placement or usage sequence, place them in instrument trays, and perform a secondary check	52	36 (69.23)	52	45 (86.53)	4.522	0.033	52	52 (100.0)	18.909	< 0.001
Inspection										
1. Request inspection personnel to double-check the quantity, model, and cleaning quality of the instruments before packaging	52	41 (78.85)	52	48 (92.31)	3.817	0.051	52	52 (100.0)	12.301	< 0.001
Packaging and sealing										
1. Examine the non-woven fabric for any damage or stains	52	30 (57.69)	52	40 (76.92)	4.370	0.037	52	50 (96.15)	21.667	< 0.001
2. Use two layers of non-woven fabric for dual packaging (parallel style or envelope style)	52	44 (84.62)	52	52 (100.0)	8.667	0.003	52	52 (100.0)	8.667	0.003
3. Ensure appropriate tension in the packaging, and use an adhesive tape of suitable length for the size of the packages	52	35 (67.31)	52	44 (84.62)	4.265	0.039	52	49 (94.23)	12.133	< 0.001
Labeling										
1. Affix the external label, ensuring clear visibility of label information and traceability of QR code	52	39 (78.85)	52	45 (86.53)	2.229	0.135	52	52 (100.0)	14.857	< 0.001
2. Check the item name, sterilization time, expiration date, packaging operator, verification personnel, and department	52	26 (50.0)	52	36 (69.23)	3.994	0.046	52	46 (88.46)	18.056	< 0.001

## Discussion

### Effectiveness of intervention measures and their impact on the occurrence of packaging defects

Figure 2 has shown that the majority of the defects occurred in instrument quantity, cleaning quality, chemical indicators and sealing. These defects were the major factors associated with packaging quality. More attention should be paid to these factors. Table 2 has shown that the incidence of packaging defects was significantly reduced after action (*P* < 0.05). Packaging plays a crucial role in the entire process of instrument handling. It entails inspecting and packaging devices once they have been cleaned and disinfected. Due to the high volume of daily surgeries and the large circulation of instruments, it is critical to ensure both speed and the correctness and quality of the assembly in packing. This places high demands on the packaging personnel. Packaging defects can be reduced by using effective methods and scientific management^12^. Instrument packaging is performed by packaging personnel. During routine packaging work, defects may occur for various reasons. We previously focused on human factors while ignoring other issues such as processes, systems, the environment, and human resource allocation, resulting in a negligible reduction in defect numbers. In this study, we combined qualitative and quantitative research methods, integrated a literature review, and conducted discussions on identified problems by the action group members through supervision to develop an intervention plan. The intervention plan was constructed using an action research framework to solve practical problems^13^. Furthermore, new issues were continuously discovered, and solutions were sought during the problem-solving process, making it highly practical^14^. The development of standardized processes is a critical component of the intervention strategy. Our study allowed packaging personnel to participate in the study, thereby ensuring that packaging personnel can effectively reduce the occurrence of packaging defects and improve their compliance with the process by implementing the packaging process. This study analyzed actual problems based on real work scenarios and aimed to improve practice quality. The intervention plan constructed has significantly improved the occurrence of packaging defects.

### Improvement schemes based on action research can enhance the proficiency of packaging personnel

Packaging is a fundamental operational skill that CSSD personnel should possess. Packaging entails more than just establishing a sterile barrier; it primarily includes assembly, packaging, sealing, labeling, and other steps. Through investigations into the actual packaging process, it was discovered that packaging personnel was unfamiliar with inspection criteria, inspections were incomplete, packaging methods lacked uniformity, and there were process discrepancies, all of which led to defects. Table 3 depicts a significant improvement in the compliance rate of process execution after two cycles of action. Table 3 has shown that the number of packaging persons who got “pass” for each step of the packaging procedures was significantly increased following the second cycle of action (*P* < 0.05). Based on action research, the intervention scheme incorporates theoretical knowledge into practice. Managers flexibly choose training methods based on actual circumstances, combining online and offline approaches supplemented by operational video demonstrations. This approach considerably improves the convenience of learning for packaging personnel. Considering the fatigue and low efficiency that might occur in learning, managers evaluated packaging personnel’s work results by normal work quality control and encouraged packaging personnel to improve their packaging skills at work, thereby avoiding the stress and time consumption caused by centralized training to the packaging personnel. Throughout the action process, group members thoroughly collect issues in their daily work. Adjustments and optimizations to the process were made by starting with real work and the perspective of packaging personnel, embodying the concept of action research, which effectively solves problems through the participation of practitioners^15^. It effectively enhances the business competence of packaging personnel while tapping into the evaluative thinking abilities of the workforce.

### Action research promotes the normalization of implementing and managing improvement plans

The qualities of action research, such as continuous evaluation and fast feedback^16^, allow for the timely revision of plans during the implementation process. It also encourages practitioners to provide novel ideas and proposals, combining the knowledge and skills of both researchers and practitioners to solve problems jointly^17^. The intervention strategy standardizes the packaging process and develops corresponding solutions in terms of management, making the packing process simpler to resolve interfering factors. Packaging personnel participates in the study as practitioners and participants, having a proactive attitude toward carrying out the strategy^18^. They continuously provide suggestions during the two implementation cycles and implement the proposed improvements, ensuring the smooth progress of the research, promoting teamwork, and facilitating collective progress.

### Limitations

This was a single-center study. The study samples only contained the instruments using closed-type packaging, so the types of the instruments were not sufficient. This might cause deviation of the research results. We hope we can have more types of samples and an enlarged sample size for our future multi-center study, thereby verifying the validity of the intervention scheme in extensive application.

## Conclusions

This study explains the application of action research methodology to improve the packaging procedures and verification contents of medical instrument packaging. Continuous questioning and timely modifications were made to reduce the defect rate and enhance the packaging quality by involving packaging personnel in the research process. The research findings confirm that the revised and refined packaging process scheme can reduce the incidence of packaging defects and improve the professional competency of packaging personnel following the 2 rounds of action research cycle. The research and implementation is reasonable, cooperative, participatory and reflective, and has good operability in improving instrument packaging quality in the CSSD. The development of the research methodology also confirms the applicability of action research in work procedures for the CSSD. Our study can provide a reference for the improvement of other issues in the CSSD.

## Data Availability

The datasets used and/or analysed during the current study are available from the corresponding author on reasonable request.

## Abbreviations

CSSD: Central sterile supply department
SOP: Standard operating procedure
